# Protocol for a Pre‐Post Field Trial of a Home Hearing and Vision Care Program for Older Australians With Diverse Cognitive Abilities and Hearing and/or Vision Impairment

**DOI:** 10.1002/hsr2.71931

**Published:** 2026-03-19

**Authors:** Melinda Toomey, Helen Gurteen, Bec Bennett, Dayna Cenin, Najwan El‐Saifi, Melanie Ferguson, Yuanyuan Gu, Chyrisse Heine, Lisa Keay, Sheela Kumaran, Sabrina Lenzen, Iracema Leroi, Judy A. Lowthian, Carly Meyer, Leander Mitchell, John Newall, Nancy A. Pachana, Marianne Piano, Smriti Raichand, Emma Scanlan, Hamid R. Sohrabi, Piers Dawes

**Affiliations:** ^1^ Center for Hearing Research, School of Health and Rehabilitation Sciences The University of Queensland Brisbane Australia; ^2^ National Acoustic Laboratories Macquarie University Sydney Australia; ^3^ Brightwater Research Center Perth Australia; ^4^ School of Population and Global Health University of Western Australia Perth Australia; ^5^ School of Allied Health Curtin University Perth Australia; ^6^ Macquarie University Center for the Health Economy, Macquarie Business School & Australian Institute of Health Innovation Macquarie University Sydney Australia; ^7^ Institute of Health and Well‐being Federation University Australia Ballarat Australia; ^8^ School of Optometry and Vision Science UNSW Sydney Sydney Australia; ^9^ Center for the Business and Economics of Health The University of Queensland Brisbane Australia; ^10^ Department of Psychiatry, School of Medicine Global Brain Health Institute, Trinity College Dublin Dublin Ireland; ^11^ School of Public Health and Preventive Medicine Monash University Melbourne Australia; ^12^ Bolton Clarke Research Institute Brisbane Australia; ^13^ School of Psychology The University of Queensland Brisbane Australia; ^14^ Department of Linguistics, Faculty of Medicine, Health and Human Sciences, The Australian Hearing Hub Macquarie University Sydney Australia; ^15^ Department of Optometry and Vision Sciences, Melbourne School of Health Sciences University of Melbourne Melbourne Australia; ^16^ National Vision Research Institute, Australian College of Optometry Melbourne Australia; ^17^ Hearing Australia Sydney Australia; ^18^ Center for Healthy Ageing Murdoch University Perth Australia; ^19^ School of Psychology Murdoch University Perth Australia; ^20^ Manchester Center for Audiology and Deafness University of Manchester UK

**Keywords:** dementia, hearing impairment, home care, vision impairment

## Abstract

**Background and Aims:**

Hearing and vision impairments are prevalent health conditions among older adults, negatively affecting functional ability, mental and social well‐being, quality of life and increasing the likelihood of transition to long‐term care. Sensory impairments are also common comorbidities with dementia, compounding the impact of dementia. Early intervention to optimize sensory function may help older adults stay in their homes longer. The Australian government's ‘Homecare Package’ provides affordable care services to support older adults with complex needs at home; however, there is a lack of support for hearing and vision needs. This trial will investigate whether a sensory support intervention enhances the quality of life and other outcomes for older adults (aged 65 years or older) with diverse cognitive abilities and hearing and/or vision impairments, as well as their care partners.

**Methods:**

This manuscript presents a study protocol for a home‐based sensory support intervention for older adults with hearing and/or vision impairment who receive home care services. A pre‐post study will determine the impact and cost‐effectiveness of a 12‐week intervention. Key outcome measures include health‐related quality of life, sensory and cognitive functional ability, relationships, mental well‐being and health resource utilization. All outcomes will be assessed before the intervention (baseline) and 13‐ and 26‐weeks post‐intervention commencement.

**Conclusion:**

This manuscript describes the protocol for a pre‐post intervention trial. A parallel and complementary process evaluation will be detailed in a separate publication. If effective, the intervention may assist older adults to improve their quality of life and remain living in their own homes. Findings will generate evidence for hearing and vision support as part of home care services.

**Trial registration:** ACTRN12624001167550 retrospectively registered.

## 1 Introduction

Hearing and vision impairments are common among community‐dwelling older adults (60 ≥ years) globally, with prevalence rates of 48.3% to 66.5% for hearing loss [1, 2] (defined as > 25dBHTL) and 10.3% to 21.8% for vision loss (defined as visual acuity < 20/40) [3, 4]. These impairments often co‐occur with dementia, which affects 18.7% to 37.1% of community‐dwelling people aged over 65 years worldwide [5, 6]. Sensory impairments can exacerbate dementia‐related declines in cognition, functional, behavior, quality of life, and independence [7, 8]. People with hearing and/or vision impairments or dementia have a greater risk of transitioning to long‐term care facilities due to reduced independence and functional ability [9, 10]. Care partners (family or friends providing support) also experience poorer mental and physical health, especially when supporting individuals with dementia or hearing and/or vision impairment [11, 12].

Hearing and vision interventions improve outcomes for older adults [13, 14], including those with dementia [13], and benefit care partners [15]. Interventions include hearing aids, communication training, spectacles, magnifiers, cataract surgery, and low vision rehabilitation. Early detection and intervention can reduce the risk of severe impairment [16, 17], yet sensory loss is often under‐detected and under‐managed [18]. The gradual onset of hearing and vision impairments often leads older adults to dismiss symptoms as normal aging, which may consequently delay treatment [19, 20]. Dementia complicates detection, as affected individuals may not recognize or communicate hearing or vision difficulties, and care partners may misattribute symptoms to dementia [3, 21].

In Australia, hearing and vision care is predominantly provided by community‐based audiologists and optometrists. Yet, limited engagement with these professionals is a key barrier impacting the health, well‐being, and independence of people receiving aged care services [22]. Barriers include perceived stigma, low awareness of rehabilitation services, accessibility challenges (particularly in rural areas) and out‐of‐pocket costs [23, 24].

Most older adults prefer to ‘age in place’ rather than relocating to long‐term care facilities [22]. Home‐based care reduces costs compared to long‐term care [25], a key priority for governments facing rising care expenses for aging populations [26]. Home care minimizes formal care expenses (accommodation, food, facility maintenance, etc), reduces hospitalizations and leverages informal supports [25]. In 2023‐24, the Australian Government spent AU$21.5 billion on long‐term aged‐care, more than double the AU$11.5 billion allocated to home care [27], with per‐person costs of AU$5,100 for long‐term aged‐care and AU$2,600 for home care [27].

The Australian Government funds home care services for people aged 65 years or older through the ‘Commonwealth Home Support Program’ and ‘Home Care Packages’, addressing basic to complex needs [28]. Services include cleaning, meal preparation, personal care, transport, social support, and nursing care. Usage has quadrupled over the last decade [29], with 24,000 additional packages funded in 2024–2025 [30].

Hearing and vision care have only recently been included in Home Care Packages, so routine checks are rare, and formal referral pathways to hearing or vision care services are lacking. Consequently, hearing and vision impairments frequently go under‐identified and under‐treated, because other care needs are prioritized, and care staff lack expertise in identifying and supporting hearing and vision needs [31]. Effective management and support of hearing and vision health in home care settings requires a multifaceted approach involving older adults, care partners, home care service providers, and the broader care ecosystem [22].

To address the need for integrated hearing and vision services in home care, a home‐delivered sensory support intervention (SSI) was developed to improve outcomes for Australians receiving home care services. Originally based on European research involving people with dementia and their care partners [32], the SSI was adapted through co‐design for broader use in Australia. Co‐design involved older adults with hearing, vision and cognitive impairments, care partners, consumer representatives, and hearing, vision and home care professionals. Tailoring ensured alignment with Australia's aged home care structure, healthcare system, and regulations [33]. The resultant SSI targets older adults with hearing and/or vision impairment, with or without cognitive impairment. This inclusive approach reflects the complex care needs of the ageing population and aims to improve outcomes for all adults receiving home care services.

This paper describes the protocol for a prospective pre‐post‐trial evaluating the SSI across three domains: (i) impact on older adults' quality of life, well‐being, functional ability, and behaviors, (ii) impact on care partners' quality of life, well‐being and relationship quality and (iii) costs and cost‐effectiveness via within‐trial analysis and economic modeling. The research questions to be answered are outlined in Table 1.

**Table 1:** Research questions.

RQ1	What is the impact of the sensory support intervention on the quality of life of home care recipients and their care partners?
RQ2	What is the impact of the sensory support intervention on home care recipients’ functional ability, visual function, hearing function, and mental and social well‐being?
RQ3	How does the cognitive status of the home care recipient mediate the outcomes of the sensory support intervention, and what are the additional supports required for people with dementia?
RQ4	What is the impact of the sensory support intervention on the mental well‐being of care partners and the quality of their relationship with the home care recipient?
RQ5	What are the costs associated with, and the cost‐effectiveness of, the sensory support intervention compared to usual care?

Abbreviation: RQ, research question.

## 2 Methods

**Figure 1:**
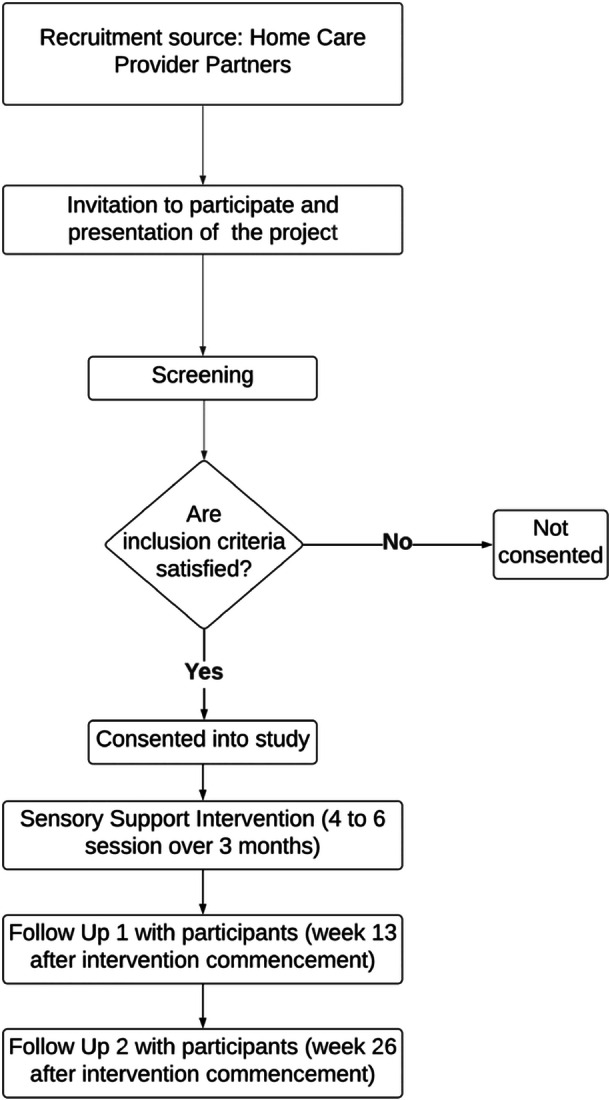
Flowchart of the study showing participant recruitment, intervention delivery, and follow‐up.

The intervention will be delivered over 3 months, with follow‐up at 13‐ and 26‐weeks post‐commencement of the intervention (Figure 1). This 26‐week duration balances participant burden with capturing both short‐ and longer‐term effects. The study protocol follows the Standard Protocol Items: Recommendations for Interventional Trials (SPIRIT) statement (Supplement 1 and 2) [34], and the intervention description, the Template for Intervention Description and Replication (TIDieR) checklist and guide (Supplement 3) [35]. A separate process evaluation protocol [36], outlines a mixed‐methods approach, guided by the UK Medical Research Council framework, to assess implementation fidelity, mechanisms of impact and contextual factors.

### 2.1 Ethical Considerations

Ethical approval was granted by The University of Queensland Human Research Ethics Committee (HE002236; 2024). Participants will receive an information sheet, discuss the study with a researcher, and provide written consent. Accessible materials will be available for participants with dementia or sensory impairments, including plain‐language explanations, large print, audio, and communication support. For deafblind participants, tactile sign language interpreters or other specialist communication methods will be arranged. Proxy consent and participant assent will be obtained where appropriate. If unable to sign, a nominated person may complete the form in the presence of a researcher.

### 2.2 Setting

This study will be conducted in the Greater Brisbane metropolitan area (Queensland, Australia) using in‐person and online data collection methods. The intervention will be delivered in participants' homes and clinic settings (audiology and optometry). Data will be collected via online surveys in Qualtrics (Qualtrics XM, Seattle, WA).

### 2.3 Participants and Sample Size

Participants will be older adults with hearing and/or vision loss, and optionally, their informal care partners. Inclusion criteria for older adults are (i) aged 65 years or older, (ii) cognitive status from normal to moderately‐advanced dementia (Functional Assessment Staging Test Score Stage 6) [37], (iii) residing at home and receiving home care services; (iv) capacity to provide informed consent or have a care partner to provide proxy consent, (v) adult‐acquired hearing impairment and/or adult‐acquired vision impairment as defined in Table 2. Sex and gender will be recorded at baseline, with recruitment monitored to support balanced representation. Individuals scheduled for cataract surgery within the study period will be excluded, as postoperative improvements in vision may affect outcome measures related to visual function and quality of life.

**Table 2:** Hearing and screening protocol and hearing and vision impairment inclusion criteria.

Screening type	Equipment used	Description	Inclusion criteria
Hearing Screening	Audiometry by HearX (hearX group, Pretoria) [38]	Each ear will be tested at pure tone frequencies of 500, 1000, 2000, 4000 and 8000 Hz.	Hearing worse than 20 dB HL at 1000 Hz or 2000 Hz, or worse than 35 dB HL, 4000 Hz, or 8000 Hz in the better hearing ear
Vision Screening	Visual acuity by Peek Acuity (Peek Vision) [39]	Snellen acuity for each eye.	Presenting monocular visual acuity of 6/12 or worse in the better eye.

The target sample size is 87 older adults, based on a repeated measures statistical model powered to detect a standardized effect of *d* = 0.3, with 80% power, and a two‐sided 5% significance level, assuming a 0.6 correlation between baseline and 13‐week, follow‐up with the Health Utility Index 3 (HUI3) scores and a 20% attrition rate [40, 41]. Consecutive sampling will be used to invite all eligible participants, thereby minimizing selection bias [42].

Care partners must be ≥ 18 years of age, able to provide informed consent, have conversational English and have regular contact (at least weekly via in‐person, phone, or video) with their care recipient, newsletters.

### 2.4 Recruitment

Eligible participants will be recruited through partner home care providers (e.g., BallyCara, Bolton Clarke, etc.). Recruitment will occur via email, newsletters, social media, community information sessions at home care provider community centers or direct contact by home care provider staff (e.g., case managers, personal care workers, nurses).

### 2.5 Screening

Screening will confirm age (≥ 65 years), current receipt of home care services, and presence of hearing and/or vision impairment (Table 2). An otoscopic examination will be conducted before the hearing screening to detect earwax. Individuals with wax will be referred for removal before assessment.

### 2.6 Intervention Description

The SSI was adapted from the European SENSE‐Cog SSI, using the UK Medical Research Council's framework for complex interventions [43]. The original intervention was field‐tested and trialed over 5 years [32, 44]. To ensure relevance in Australia [33], the SSI was adapted through iterative co‐design involving home care recipients with hearing and/or vision impairment, care partners, and hearing, vision and home care professionals. This process identified unmet hearing and vision support needs [45], and then adapted the intervention to those needs via a series of interviews and workshops. A Patient and Public Involvement (PPI) group with lived experience of hearing, vision and cognitive impairment reviewed and refined the adapted SSI to ensure suitability for older Australians with hearing and/or vision impairments.

The SSI has been designed to accommodate older adults with single or dual sensory impairment. The intervention includes screening and referral to community clinicians for audiometric or optometric correction, supporting effective assistive device use (e.g., hearing aids), environmental modification (e.g., increasing lighting levels), accessing relevant support services (e.g., low vision services), enhancing communication between the participant and care partner (e.g., communication training), and promoting social inclusion (e.g., connecting with local interest groups). Components are tailored to an individual's hearing and/or vision needs, cognitive and physical ability, current knowledge and skills, access to services, environmental factors, and personal preferences. The intervention comprises eight components (Table 3). Components A, B and C are delivered first, while D to H are delivered flexibly based on participant needs. Key components (A to E) are delivered to all participants, whilst optional components (F to H) are offered based on individual needs and preferences.

**Table 3:** Description of the sensory support invention components with examples of modifications from co‐design and how the component might be tailored to the individual.

Component	Description (Component Type and Duration)	Example Modifications from Co‐design	Example of Tailoring
A. Identify any vision and hearing concerns and discuss support options	Key Component, duration 60 min The Sensory Therapist discusses hearing and vision concerns and support options with participants, develops a Personal Sensory Plan and arranges hearing and/or vision assessments.	Converted the Personal Sensory Plan questionnaire into an online survey with branching logic to recommend the components that would address needs identified.	For a participant with dual sensory impairment, the Sensory Therapist uses amplification and written materials in large print with high contrast during discussions.
B. Home‐based functional assessment and goal setting	Key Component, duration 120 min A functional assessment will be conducted to ascertain how the home care recipient's cognitive ability and sensory impairments impact a broad range of daily living activities. The assessment outcomes will help shape other components of the sensory support intervention, including referral for support, social inclusion, and supplementary devices. The Sensory Therapist will incorporate the results of the hearing and/or vision assessments and the functional assessment results to help the participants and their study partner set personalized goals using the Bangor Goal Setting Inventory [46]. Goals will be revisited at each visit and the Sensory Therapist will explore facilitators, barriers and resources to the goals and introduce skills and strategies to support progress.	Enhanced the Functional Assessment checklist with representative images to improve accessibility for people with dementia or who are culturally and linguistically diverse.	For participants with cognitive impairment, a pictorial decision tool is used during goal setting to support understanding and choice‐making.
C. Optimization of any vision or hearing impairment	Key Component, duration 60 min An audiologist or optometrist will undertake vision and hearing assessments according to standard procedures as required. The Sensory Therapist helps arrange transport to and from hearing and/or vision appointments with family and/or home care providers. The participant's usual optometrist will provide optometric services. Participants will be reimbursed for the cost of their spectacles up to a $AU 250 amount. Hearing Australia will provide audiological services. Starkey Hearing Technologies and Hearing Australia will provide hearing aids free of charge for the study.	Incorporated a link to Optometry Australia's ‘Find an Optometrist’ feature that allows users to search for dementia‐friendly or low vision specialists in their local area.	For a participant with mobility limitations and no family support, the Sensory Therapist might organize a mobile service to provide a domiciliary visit, with a longer appointment time to accommodate a slower processing speed.
D. Continuous training in correct use of sensory devices	Key Component, duration 60 min The Sensory Therapist will work with participants to enhance adherence and correct usage of the prescribed hearing aids and spectacles. This will include advice given on wearing times, handling, maintenance, cleaning, and storage of devices. The following tests will be administered to monitor participants’ ability to manage their sensory equipment: Hearing Aid Skills and Knowledge test [47] and SENSE‐Cog Glasses Skills and Knowledge test for vision [48].	Hearing aid user and glasses user guides were developed that featured commonly dispensed Australian products.	A participant with arthritis and mild dementia receives simplified, step‐by‐step visual guides for hearing aid insertion and extended hands‐on practice sessions. Training is broken into shorter sessions with frequent repetition, while the study partner is taught to provide daily prompts and assistance.
E. Communication training	Key Component, duration 60 min The Sensory Therapist will work with participants to enhance communication between the home care recipient and their family member/informal caregiver using the SENSE‐cog Communication Manual, which was adapted from existing evidence‐based resources relating to hearing and/or vision impairment and dementia to provide guidance and strategies to enhance communication in different settings. The participants will be provided copies of pre‐existing materials, such as leaflets and video‐based training.	Online Communication Tactic Tool using branching scenarios was developed to provide personalized recommendations.	For a couple where the care recipient has both hearing loss and cognitive impairment, training emphasizes getting attention before speaking, using shorter sentences, and allowing extra processing time. The study partner learns to reduce background noise during conversations and verify understanding without causing frustration.
F. Referral to health and social services	Optional Component, duration 20 min Based on the functional assessments and goals set in Component B, the Sensory Therapist may refer participants to community health or social care services (e.g., psychological services, low vision services, geriatric psychiatry services, falls clinic, etc.)	A list of Australian health and social services, including consumer advocacy groups (e.g., Older Persons Advocacy Network, Vision Australia, Better Hearing Australia, etc.) was created.	A participant experiencing social withdrawal and low mood following vision loss is referred to a low vision support group and counseling services, with the Sensory Therapist facilitating initial contact and providing information in large print format.
G. Fostering social inclusion through hobbies/interests/social groups	Optional Component, duration 20 min In line with the participants’ goals set in component B, the Sensory Therapist will provide information and guidance on opportunities to develop their hobbies and interests or attend local social groups.	Developed a Hobby and Activities decision guide with links to events and activities in Brisbane and surrounds.	A former book club member with vision loss is connected with a local audio book group and provided with information about accessible library services and large‐print book delivery programs that align with their cognitive abilities and reading interests.
H. Environmental modifications and assistive devices	Optional Component, duration 20 min Based on the functional assessment and goals set in component B, the Sensory Therapist may arrange and support the use of environmental modifications (e.g., lighting, increasing contrast, reducing reverberation) or assistive devices (e.g., magnifiers, frequency modulation systems).	Compilation of smartphone accessibility tools (e.g., live captioning) and apps (Be My Eyes) that the Sensory Therapist could recommend to participants was developed.	For a participant with vision impairment and cognitive decline living in a poorly lit home, modifications include installing motion‐sensor lighting in hallways, high‐contrast tape on stairs, and a large‐button amplified telephone. The complexity of devices is matched to the participant's cognitive capacity for independent use.

Delivered over 3 months in 4–6 home‐based sessions (1–2 h each), the intervention is led by a specialist‐trained Sensory Therapist. Training comprises didactic instruction from an audiologist and optometrist, plus an online program covering hearing and vision rehabilitation, communication strategies, assistive technologies, and adaptation to recognize and respond to the needs and challenges of cognitive and dual sensory impairment.

The Sensory Therapist will develop an individualized Personal Sensory Plan (Component A) using bespoke online tools to assess the sensory needs, preferences and functional ability of the participant. The Personal Sensory Plan provides specific recommendations and intervention components to meet those needs. If hearing or vision optimization is needed (i.e., new glasses or hearing aids, or updating existing assistive devices), participants will be referred to an audiologist or optometrist (Component C). Participants then engage in goal‐setting using the Bangor Goal Setting Inventory to prioritize sensory needs [46]. Intervention components are tailored based on hearing and/or vision function, cognitive ability and participant goals. Tailoring is determined collaboratively between the Sensory Therapist, participant and care partner.

All intervention materials are hosted on Canvas (Canvas Instructure, Salt Lake City, UT) an online Learning Management System with accessibility features including black‐on‐white text, high contrast colors, 14‐point font size, text descriptions for images, descriptive hyperlinks, text‐to‐audio functionality and a clear, consistent user interface. The Sensory Therapist will demonstrate platform navigation to participants, who are encouraged to review the information and resources between sessions to consolidate their knowledge and skills. For those without internet access or the skills to use online devices, accessible paper‐based resources will be provided.

### 2.7 Data Collection

Quantitative outcomes will be captured at baseline and at 13‐ and 26‐weeks post‐intervention commencement using self‐ or proxy‐report questionnaires (online, paper format, or phone‐administered) (Table 4). Questionnaires take 70 min to complete for home care recipients and 55 min for care partners. For participants living with dementia, accommodations include proxy completion by a care partner, support from a care partner during interviews, and reframing questions to suit communication abilities [49].

**Table 4:** Overview of the time schedule of intervention and outcome measures.

	Eligibility Screening	Baseline	Intervention Period	Follow‐up		
Timepoint	W‐4–W0	W0	W1–W12	W13	W14–W17	W26
Screening						
Eligibility screening	X					
Informed consent		X				
Intervention						
Sensory Support Intervention						
Assessments						
Home Care Recipient						
Demographic and health status		X				
Sensory and cognitive status		X				
Hearing Screening						
Automated audiometry screening (HearX)	X					
Otoscopy	X					
Vision screening						
Visual acuity screened by Peak Acuity	X					
Cognition screening						
MoCA – HI/VI		X				
FAST Tool	X					
QoL, Function, Well‐being						
HUI3		X		X		X
QOL‐ACC		X		X		X
ASCOT (SCT‐4)		X		X		X
IADLS		X		X		X
RHHIE‐S		X		X		X
NEI VFQ‐25		X		X		X
GAI‐SF		X		X		X
GDS‐5		X		X		X
NPI‐Q		X		X		X
UK Biobank – Social interaction		X		X		X
Care Partner						
Demographic and health status		X				
EQ‐5D‐5L		X		X		X
GAI‐SF		X		X		X
GDS‐5		X		X		X
MoCA		X		X		X
RSS		X		X		X
FCRS		X		X		X
SOS‐HEAR		X		X		X
Resource use						
RUD‐Lite		X		X		X

Abbreviations: ASCOT (SCT‐4), Adult Social Care Outcomes Toolkit 4; EQ‐5D‐5L, European Quality of Life 5‐dimension 5‐level; FAST, Functional Assessment Staging Tool; FCRS, Family Caregiving Role Scale; GAI‐SF, Geriatric Anxiety Inventory Short Form; GDS‐5, Geriatric Depression Scale 5‐item; HUI3, Health Utility Index 3; IADLS, Instrumental Activities of Daily Living Scale; MoCA, HK/VI Montreal Cognitive Assessment Hearing Impaired or Vision Impaired; NEI‐VFQ‐25, National Eye Institute Visual Function Questionnaire 25 item; NPI‐Q, Neuropsychiatric Inventory Questionnaire; QOL‐ACC, Quality of Life Aged Care Consumer; RHHIE‐S, Revised Hearing Handicap Inventory for the Elderly Screening version; RSS, Relationship Satisfaction Scale; RUD‐LITE, Resource Utilization in Dementia Lite; SOS‐HEAR, Significant Other Scale for Hearing Disability; W, week.

### 2.8 Data Management Plan

Data will be securely stored on The University of Queensland's Research Data Management System, accessible only to the research team. Paper data will be digitized, then securely destroyed. Personal identifiers will be replaced with participant codes, with the password‐protected linking file deleted 6 months post‐study. With consent, non‐identifiable data will be archived in a public repository per the National Health and Medical Research Council's Open Access Policy [50] and FAIR Principles [51].

### 2.9 Outcome Measures

Participant information will include sociodemographic, health status, medication use, co‐morbidities, healthcare utilization, duration of sensory loss, and key aged care metrics. The feasibility of the number of outcome measures has been confirmed in previous trials with a similar number of outcome measures, which demonstrated high completion rates (76%) [44].

#### 2.9.1 Primary Outcomes

Primary outcomes are the home care recipient's self‐rated quality of life, measured using the HUI3 [52] and Quality of Life – Aged Care Consumer (QOL‐ACC) [53] (Table 5). HUI3 is a 40‐item interviewer‐administered (self‐report and proxy) questionnaire that assesses eight health attributes over 1‐week: vision, hearing, speech, ambulation, dexterity, emotion, cognition, and pain [52]. HUI3 has good psychometric properties [52], and has been validated for people with hearing impairment [70], and home care populations [71]. The HUI3 captures quality of life related to hearing and vision and, therefore, may be sensitive to the effect of the intervention in improving health‐related quality of life. The QOL‐ACC, a 6‐item questionnaire assessing mobility, pain, emotional well‐being, independence, social connections, and activities, has been validated for older adults in home care, including those with dementia [53].

**Table 5:** Battery of Outcome Measures with Scoring Information Administered During Baseline, Week 13, and Week 26.

Construct	Time	Administered by	Information about	Research Question Addressed	Instrument	Description	Scoring & Interpretation
Global Cognitive Functioning	BL	Researcher	HCR Caregiver		Montreal Cognitive Assessment (MoCA) (hearing impaired [54] or vision impaired [55] versions as appropriate	MoCA: 30 items assessing multiple cognitive domains. In the MoCA Hearing Impairment, the items that are presented in spoken format are substituted with suitably adapted written items. Likewise, in the MOCA Vision Impairment, the items that are presented in written format are substituted with adapted verbal items.	The MoCA has a maximum score of 30 points. A final score of 26 and above is considered normal.
	BL		HCR		Functional Assessment Staging Tool (FAST) [37]	The FAST scale is a functional scale designed to evaluate patients with dementia.	The FAST stages dementia from levels 1 to 7, with level 1 representing a normal adult and 7 representing severe dementia.
Health‐Related Quality of Life	BL, W13, W26	Researcher	HCR	RQ1, RQ5	Health Utilities Index Mark 3 (HUI3) [52]	40‐item questionnaire interviewer‐administered (self‐report and proxy) that is a multi‐attribute utility instrument used to measure health‐related quality of life over a 1‐week period. It evaluates the attributes of vision, hearing, speech, ambulation, dexterity, emotion, cognition, and pain.	HUI3 multi‐attribute utility score scales scores from −0.36 to 1.00, where 0 = dead and negative worse than dead and 1 = perfect health. Differences of 0.03 are clinically important. HUI3 single attribute utility scale scores from 0.00 to 1.00. Differences of 0.05 are clinically important.
	BL, W13, W26	Researcher	HCR	RQ1, RQ5	Quality of Life – Aged Care Consumer (QOL‐ACC) instrument [56]	6‐item questionnaire that incorporates dimensions important and preferred by older people in aged care services. It evaluates mobility, pain, emotional well‐being, independence, social connections and activities.	The QOL‐ACC has a maximum score of 24, where ‘excellent’ quality of life = 22–24, ‘good’ = 19–21, ‘moderate’ = 14–18, ‘poor’ = 8–13, and ‘very poor’ = 0–7.
	BL, W13, W26	Self‐reported	Caregiver	RQ4	European Quality of Life 5‐dimension 5‐level (EQ‐5D‐5L) [57]	5‐item self‐report questionnaire describing five dimensions: mobility, self‐care, usual activities, pain/discomfort, and anxiety/depression	Scores recorded for each test item will be indexed based on the Australian value set. Participant's health related quality of life will be described for each of the five dimensions, and quantified as either; no problems, slight problems, moderate problems, severe problems, or extreme problems
Social Care‐Related Quality of Life	BL, W13, W26	Researcher	HCR	RQ2	Adult Social Care Outcomes Toolkit (SCT4) [58]	9‐item self‐reported questionnaire that is a multi‐attribute utility instrument that captures aspects of social care‐related quality of life.	The SCT4 scores between −0.17 to 1.00, where 0 = dead and negative as worse than dead and 1 = ideal state.
Quality & Quantity of Social Interactions	BL, W13, W26	Researcher	HCR	RQ2	Questions from UK Biobank [59]	3‐item self‐reported questionnaire that assesses social interaction	The Biobank scores for social isolation range from 0 to 3, where 0 = least isolation, 1 = moderate isolation, ≥ 2 = most isolation.
Functional Ability	BL, W13, W26	Researcher	HCR	RQ2	Instrumental Activities of Daily Living Scale [60]	8‐item questionnaire that assesses independent living skills.	Each item is scored as 1 = independent and 0 = dependent. Therefore, the final score ranges from 0 = low function, dependent to 8 = high function, independent.
Vision Function	BL, W13, W26	Researcher	HCR	RQ2	National Eye Institute Visual Function Questionnaire 25 item (NEI VFQ‐25) [61]	25‐item self‐reported measure of vision‐related functioning.	Each item is scored from zero to 100 (best level) so that a high score indicates good quality of life. The composite NEI VFQ‐25 is the mean score of all items except for the general health item.
Hearing Function	BL, W13, W26	Researcher	HCR	RQ2	Revised Hearing Handicap Inventory for the Elderly Screening version [62]	10‐item self‐report measure that asks about the emotional impacts of hearing loss.	The RHHI‐S scores between 0 to 40, where 0 = no handicap and 40 = maximum handicap.
Anxiety	BL, W13, W26	Researcher Self‐reported	HCR Caregiver	RQ2, RQ4	Geriatric Anxiety Inventory (GAI) Short Form [63]	5‐item self‐reported questionnaire that assesses anxiety in older adults.	The GAI‐SF has a maximum score of 5. A score of 3 and above indicates the presence of clinically significant anxiety.
Depression	BL, W13, W26	Researcher Self‐reported	HCR Caregiver	RQ2, RQ4	Geriatric Depression Scale SF (GDS‐5) [64]	5‐item self‐reported questionnaire that assesses depressive symptoms in elderly people	The GDS‐SF has a maximum score of 5. A score of 2 and above indicates possible depression.
Dementia Symptoms	BL, W13, W26	Proxy Reported	HCR	RQ2	Neuropsychiatric Inventory Questionnaire (NPI‐Q) [65]	12‐item self‐administered questionnaire completed by informants about a person for whom they care. It provides a brief assessment of neuropsychiatric symptoms experienced by the patient by exploring 12 domains	The initial response to each domain question is Yes (present) or No (absent). If Yes, the informant then rates both severity of symptoms (3‐point scale) and the associated impact of the symptoms on the carer (6‐point scale).
Health Resources Used	BL, W13, W26	Self‐reported	Caregiver	RQ5	Resource Utilization in Dementia (RUD) Lite [66]	The RUD‐lite is a standardized instrument that measures data on formal and informal care resource use, using the caregiver as the informant.	Not scored
Quality of Relationship	BL, W13, W26	Self‐reported	Caregiver	RQ4	Relationship Satisfaction Scale (RSS) [67]	7‐item self‐reported questionnaire about communication and openness, resolving conflicts and arguments and the degree of affection and caring.	The RSS scores are between 0 to 42. Higher scores indicate greater satisfaction with relationship.
	BL, W13, W26	Self‐reported	Caregiver	RQ4	Family Caregiving Role Scale [68]	16‐item self‐reported questionnaire that assesses feelings related to care provision across three sub‐scales: (1) satisfaction with the caring role, (2) resentment, and (3) anger.	Mean scores are calculated for each subscale, with higher scores indicating greater satisfaction with the caring role and greater feelings of resentment and anger.
Third‐party Hearing Loss‐Related Quality of Life	BL, W13, W26	Self‐reported	Caregiver	RQ4	Significant Other Scale for Hearing Disability (SOS‐HEAR) [69]	27‐item scale that measures third‐party hearing loss‐related QoL in spouses of people with hearing loss. Measures the effects of hearing impairment on the significant other in the following domains: Changes to communication; Communication burden; Relationship changes; Going out and socializing; Emotional reactions to adaptations; Concern for partner.	Higher SOS‐HEAR scores indicate greater difficulties experienced by the carer.

Abbreviations: BL, baseline; HCR, Home Care Recipient; HL, hearing loss; RQ, Research question; S, Screening; W, Week.

Resource utilization costs will be measured using the Resource Utilization in Dementia (RUD)‐Lite questionnaire [66], which captures resource use by caregivers of people with dementia in community settings, assessing caregivers' time, hospital care and outpatient visits [66]. Care partner quality of life will be measured using the European Quality of Life (EQ‐5D‐5L) self‐rated questionnaire, covering five health domains with five severity levels [57]. Higher scores reflect poorer quality of life.

#### 2.9.2 Secondary Outcomes

Secondary outcome measures are described in Table 5 and explore hearing, vision and mental health functionality.

### 2.10 Data Analysis

#### 2.10.1 Impact Analysis

Quantitative data will be analyzed using SPSS version 29 (IBM Corp., Armonk, NY, USA). Continuous variables will be summarized as means and standard deviations (normal distribution) or medians with interquartile ranges (non‐normal). Categorical variables will be reported as frequencies and percentages. All tests will be two‐tailed, with significance at *p* < 0.001, consistent with the sample size calculation. Adjustments for multiple comparisons will be applied where appropriate, and 95% confidence intervals reported per SAMPL guidelines [72].

Multilevel regression models will assess intervention effects at baseline, 13 weeks, and 26 weeks, accounting for repeated measures and within‐subject correlations over time. These models are appropriate given the longitudinal structure of the data. Generalized linear models (GLMs) will analyze outcomes related to RQ1, RQ2, RQ3, & RQ4 based on data distribution and scale. Continuous outcomes (e.g., QOL‐ACC, NEI VFQ‐25, RSS, SOS‐HEAR) will use GLMs with an identity link function. Count outcomes (e.g., GAI‐SF, GDS‐5, NPI‐Q symptom counts) will use GLMs with a log link function with Poisson or negative binomial distribution depending on dispersion. Ordinal categorical outcomes (e.g., UK Biobank social interaction scores) will use ordinal logistic regression with a logit link function.

Covariates include demographics, health, cognitive and sensory function, sex, gender, and sensory support use. Missing data will be handled using complete case analysis, as minimal missingness is anticipated. Acute events (e.g., hospitalization, bereavement) will be documented, and sensitivity analyses excluding affected participants will assess the robustness of findings. Analyses will follow best practice reporting standards for statistical methods in biomedical research [73].

Acute or life events (e.g., hospitalization, bereavement, health crises) that may influence outcomes will be documented throughout the study. Sensitivity analyses will be conducted, excluding participants with major acute events, to assess the robustness of findings.

#### 2.10.2 Cost‐Effectiveness

Cost‐effectiveness analysis will be conducted from the healthcare system perspective, with the primary outcome being the incremental cost‐effectiveness ratio (ICER) compared with Australia's implicit cost‐effectiveness threshold [74]. Analyses will comprise a within‐trial analysis, economic modeling, and a budget impact analysis, reported in line with the Consolidated Health Economic Evaluation Reporting Standards (CHEERS) checklist [75].

Per‐patient costs and health outcomes will be assessed. Costs will comprise those associated with the SSI and healthcare resource use, ascertained via a micro‐costing study. Health outcome will be measured as quality‐adjusted life years (QALYs), combining health‐related quality of life (HRQoL; measured as health state utilities) with the time spent in each health state. HRQoL will be assessed using the HUI‐3 and QOL‐ACC for care recipients and EQ‐5D‐5L for care partners. Australian algorithms will be used to estimate the health state utilities from the QOL‐ACC [53] and HUI3 [76] responses.

The ICER will be estimated as the difference between the costs of SSI and usual care (numerator), divided by the difference between QALYs under SSI and usual care (denominator), using GLMs accounting for data distribution and covariates. Sampling uncertainty will be handled by non‐parametric bootstrap sampling with results plotted on a cost‐effectiveness plane and used to construct cost‐effectiveness acceptability curves. Uncertainty around lifetime cost‐effectiveness results will be explored using deterministic and probabilistic sensitivity analyses.

The within‐trial analysis will present unadjusted ICER over the study duration, presented as incremental costs per QALY gained by the SSI intervention compared with usual care. Economic modeling (e.g., Markov modeling) will provide an ICER that forecasts the future costs and potential HRQoL benefits over a lifetime time horizon. All costs and outcomes will be discounted in line with the National Institute of Health and Care Excellence (NICE) guidelines for health technology assessment [77]. Model structure will be informed by a literature review. Budget impact analysis will determine the financial implications of SSI implementation for federal and/or state governments over a relevant time horizon.

## 3 Discussion

This study aims to address an important gap by evaluating the impact and cost‐effectiveness of a home‐delivered intervention designed to meet the hearing and vision support needs of older Australians receiving home care services. The intervention may assist older adults with hearing and/or vision impairments to continue aging in place rather than moving into long‐term care. Further, the study will examine how the program impacts the quality of life of care partners and their relationships with their home care recipients. In line with European findings, it is hypothesized that home care recipients who access the SSI will have a higher quality of life and sensory functional ability at 3 months post‐intervention compared to pre‐intervention [32, 44].

A recent national review highlighted the need for improved hearing and vision care for older Australians receiving aged care [22], yet no previous research has examined how to support those needs within home care settings. This study will address this gap and aligns with the World Health Organization's Integrated Care for Older People (ICOPE) framework, which emphasizes person‐centered care to maintain intrinsic capacity and functional ability [78]. The SSI operationalises these principles by focusing on sensory capacity, an often overlooked domain within home care, while also personalizing care to the individual's cognitive abilities.

This research is timely, as the Australian Government is reforming home care services in 2025. The ‘Support at Home Program’, which will replace the current ‘Home Care Packages’, aims to maintain older adults' independence through access to allied health professionals, assistive technology, and home modifications [79]. By integrating hearing and vision support into home care services, this study will provide important evidence to inform service delivery options and may offer a model for enhancing functional ability and quality of life in line with both national reforms and global healthy ageing strategies.

The development of the intervention through co‐design with home care recipients, an interdisciplinary team of hearing and vision clinicians, aged care providers and members of the PPI group with lived experience, ensures that the intervention considers and meets the needs of older Australians with hearing and vision impairments. The accompanying process evaluation of the intervention described elsewhere will provide contextual insights into the experiences of participants, which will complement the results of this study and allow for further refinement of the intervention before future scaling [36]. Making the intervention materials available online or in paper format makes the intervention accessible in the preferred format of participants and may facilitate engagement by allowing them to review the information at their convenience.

Our study has some limitations. The non‐randomized pre‐post design without a control group will limit the understanding of causality and the effectiveness of the intervention. The pre‐post‐intervention design was chosen because randomized controlled trials are less suitable for complex interventions [80], and recruiting older adults is challenging and limits the number of available participants [81]. The proposed design allows evaluation in a real‐world setting, eliminating the need for larger samples typically required by other experimental designs. It is also ethically preferable to a randomized controlled design as it avoids withholding a potentially beneficial intervention from control participants [82]. Finally, implementation in a metropolitan location may limit the generalizability to rural and regional communities in Australia.

In conclusion, the results of this study will facilitate further refinement of the intervention and development of a business case for integrating hearing and vision support into home care. Future research may explore the applicability of the intervention in different settings (e.g., regional) and populations. The findings will inform aged care policy and resource allocation to help older Australians live independently and healthily at home.

Version/Date: Version 10/Amendment 6 (14/04/2025).

## Author Contributions

Piers Dawes conceptualized the study. Piers Dawes, Bec Bennett, Melanie Ferguson, Yuanyuan Gu, Chyrisse Heine, Lisa Keay, Sheela Kumaran, Sabrina Lenzen, Iracema Leroi, Judy A. Lowthian, Carly Meyer, Leander Mitchell, John Newall, Nancy A. Pachana, Marianne Piano, Emma Scanlan and Hamid R. Sohrabi are responsible for the rationale, grant proposal and funding acquisition. All authors were involved in the study design. Melinda Toomey and Helen Gurteen planned the quantitative and qualitative analyses, and Yuanyuan Gu and Smriti Raichand planned the cost‐effectiveness analyses. Melinda Toomey is responsible for project coordination and implementation. The manuscript was drafted by Melinda Toomey, while all authors provided a critical review of the paper. All authors read and approved the final manuscript.

## Ethics Statement

This study protocol was reviewed and approved by The University of Queensland Human Research Ethics Committee, approval number 2023/HE002236.

## Consent to Participate Statement

Written consent will be obtained from participants or from their proxy (e.g., people with dementia). Assent will be gained from people with dementia.

## Conflicts of Interest

The authors declare no conflicts of interest.

## Trial Coordination Statement

The study is coordinated by the University of Queensland research team, with day‐to‐day management overseen by the Chief Investigator and co‐investigators. There is no independent steering or adjudication committee; oversight is provided through regular team meetings, institutional ethics review, and adherence to UQ research governance processes.

## Transparency Statement

The lead author Melinda Toomey, Piers Dawes affirms that this manuscript is an honest, accurate, and transparent account of the study being reported; that no important aspects of the study have been omitted; and that any discrepancies from the study as planned (and, if relevant, registered) have been explained.

## Supporting information

01 Supplements.

## Data Availability

The de‐identified datasets generated and/or analyzed during this study will be made publicly available via The University of Queensland Research Data Manager (UQ RDM).
