# Supplementary material for: Protocol for a Pre‐Post Field Trial of a Home Hearing and Vision Care Program for Older Australians With Diverse Cognitive Abilities and Hearing and/or Vision Impairment
Source: Health Sci Rep. 2026 Mar 19;9(3):e71931. doi: 10.1002/hsr2.71931 (PMC13098140; doi:10.1002/hsr2.71931)
Supplement: Supplementary file 1 — 01 Supplements. [file HSR2-9-e71931-s001.docx]

# Supplement 1

SPIRIT Checklist 2025 (Hróbjartsson, Boutron et al. 2025)

| **Section/topic** | **No** | **SPIRIT 2025 checklist item description** | **Response** |
| --- | --- | --- | --- |
| **Administrative information** | | | |
| Title and structured summary | 1a | Title stating the trial design, population, and interventions, with identification as a protocol | Title page |
|  | 1b | Structured summary of trial design and methods, including items from the World Health Organization Trial Registration Data Set | Supplement 2 |
| Protocol version | 2 | Version date and identifier | Page 32, line 487 |
| Roles and responsibilities | 3a | Names, affiliations, and roles of protocol contributors | Title page |
|  | 3b | Name and contact information for the trial sponsor | Supplement 2 |
|  | 3c | Role of trial sponsor and funders in design, conduct, analysis, and reporting of trial; including any authority over these activities | Page 3, Lines 74 - 79 |
|  | 3d | Composition, roles, and responsibilities of the coordinating site, steering committee, endpoint adjudication committee, data management team, and other individuals or groups overseeing the trial, if applicable | Trial Coordination Statement, Page 3, Lines 91-96. |
| **Open science** | | | |
| Trial registration | 4 | Name of trial registry, identifying number (with URL), and date of registration. If not yet registered, name of intended registry | Supplement 2 |
| Protocol and statistical analysis plan | 5 | Where the trial protocol and statistical analysis plan can be accessed | Data Analysis: Pages 28-29, Lines 390-438 |
| Data sharing | 6 | Where and how the individual de-identified participant data (including data dictionary), statistical code, and any other materials will be accessible | Data Management Plan: Page 21, Lines 343 – 351. |
| Funding and conflicts of interest | 7a | Sources of funding and other support (eg, supply of drugs) | Funding Sources: Page 3, Lines 73 - 78 |
|  | 7b | Financial and other conflicts of interest for principal investigators and steering committee members | Conflict of Interest Statemen: Page 3, Lines 79 - 80 |
| Dissemination policy | 8 | Plans to communicate trial results to participants, healthcare professionals, the public, and other relevant groups (eg, reporting in trial registry, plain language summary, publication) | Not reported |
| **Introduction** | | | |
| Background and rationale | 9a | Scientific background and rationale, including summary of relevant studies (published and unpublished) examining benefits and harms for each intervention | Introduction: Pages 5 - 9 |
|  | 9b | Explanation for choice of comparator | N/A to study design |
| Objectives | 10 | Specific objectives related to benefits and harms | Table 1: Page 9 |
| **Methods: Patient and public involvement, trial design** | | | |
| Patient and public involvement | 11 | Details of, or plans for, patient or public involvement in the design, conduct, and reporting of the trial | Page 14, Lines 287 - 290 |
| Trial design | 12 | Description of trial design including type of trial (eg, parallel group, crossover), allocation ratio, and framework (eg, superiority, equivalence, non-inferiority, exploratory) | N/A to study design |
| Methods: Participants, interventions, and outcomes | | | |
| Trial setting | 13 | Settings (eg, community, hospital) and locations (eg, countries, sites) where the trial will be conducted | Page 12, Lines 247 - 250 |
| Eligibility criteria | 14a | Eligibility criteria for participants | Page 12-13, Lines 252 -263 |
|  | 14b | If applicable, eligibility criteria for sites and for individuals who will deliver the interventions (eg, surgeons, physiotherapists) | N/A |
| Intervention and comparator | 15a | Intervention and comparator with sufficient details to allow replication including how, when, and by whom they will be administered. If relevant, where additional materials describing the intervention and comparator (eg, intervention manual) can be accessed | Pages 13 - 17 |
|  | 15b | Criteria for discontinuing or modifying allocated intervention/comparator for a trial participant (eg, drug dose change in response to harms, participant request, or improving/worsening disease) | N/A |
|  | 15c | Strategies to improve adherence to intervention/comparator protocols, if applicable, and any procedures for monitoring adherence (eg, drug tablet return, sessions attended) | Not reported |
|  | 15d | Concomitant care that is permitted or prohibited during the trial | N/A |
| Outcomes | 16 | Primary and secondary outcomes, including the specific measurement variable (eg, systolic blood pressure), analysis metric (eg, change from baseline, final value, time to event), method of aggregation (eg, median, proportion), and time point for each outcome | Pages 21 - 27 |
| Harms | 17 | How harms are defined and will be assessed (eg, systematically, non-systematically) | Not reported |
| Participant timeline | 18 | Time schedule of enrolment, interventions (including any run-ins and washouts), assessments, and visits for participants. A schematic diagram is highly recommended. | Table 3, pages 19 - 20 |
| Sample size | 19 | How sample size was determined, including all assumptions supporting the sample size calculation | Page 12 – 12, Pages 251 - 272 |
| Recruitment | 20 | Strategies for achieving adequate participant enrolment to reach target sample size | Page 13, Lines 273 - 277 |
| **Methods: Assignment of interventions** | | | |
| Randomisation: |  |  |  |
| Sequence generation | 21a | Who will generate the random allocation sequence and the method used | N/A to study design |
|  | 21b | Type of randomisation (simple or restricted) and details of any factors for stratification. To reduce predictability of a random sequence, other details of any planned restriction (eg, blocking) should be provided in a separate document that is unavailable to those who enrol participants or assign interventions | N/A to study design |
| Allocation concealment mechanism | 22 | Mechanism used to implement the random allocation sequence (eg, central computer/telephone; sequentially numbered, opaque, sealed containers), describing any steps to conceal the sequence until interventions are assigned | N/A to study design |
| Implementation | 23 | Whether the personnel who will enrol and those who will assign participants to the interventions will have access to the random allocation sequence | N/A to study design |
| Blinding | 24a | Who will be blinded after assignment to interventions (eg, participants, care providers, outcome assessors, data analysts) | N/A to study design |
|  | 24b | If blinded, how blinding will be achieved and description of the similarity of interventions | N/A to study design |
|  | 24c | If blinded, circumstances under which unblinding is permissible, and procedure for revealing a participant’s allocated intervention during the trial | N/A to study design |
| Methods: Data collection, management, and analysis | | | |
| Data collection methods | 25a | Plans for assessment and collection of trial data, including any related processes to promote data quality (eg, duplicate measurements, training of assessors) and a description of trial instruments (eg, questionnaires, laboratory tests) along with their reliability and validity, if known. Reference to where data collection forms can be accessed, if not in the protocol | Data collection: Page 14 – 18. Lines 330 – 338  Description of instruments: Table 4, pages 23 - 27 |
|  | 25b | Plans to promote participant retention and complete follow-up, including list of any outcome data to be collected for participants who discontinue or deviate from intervention protocols | Not reported |
| Data management | 26 | Plans for data entry, coding, security, and storage, including any related processes to promote data quality (eg, double data entry; range checks for data values). Reference to where details of data management procedures can be accessed, if not in the protocol | Data collection: Page 14 – 18. Lines 330 – 338  Data management plan: Page 21,Lines 343 - 351 |
| Statistical methods | 27a | Statistical methods used to compare groups for primary and secondary outcomes, including harms | Pages 28 - 30 |
|  | 27b | Definition of who will be included in each analysis (eg, all randomised participants), and in which group | N/A to study design |
|  | 27c | How missing data will be handled in the analysis | Pages 28 - 30 |
|  | 27d | Methods for any additional analyses (eg, subgroup and sensitivity analyses) | Pages 28 - 30 |
| **Methods: Monitoring** | | | |
| Data monitoring committee | 28a | Composition of data monitoring committee (DMC); summary of its role and reporting structure; statement of whether it is independent from the sponsor and funder; conflicts of interest and reference to where further details about its charter can be found, if not in the protocol. Alternatively, an explanation of why a DMC is not needed | A Data Monitoring Committee was not established because this is a non-randomised, pre–post intervention study with minimal risk procedures. Trial oversight is maintained through institutional ethics approval, adherence to research governance requirements, and regular investigator team review of study progress.” |
|  | 28b | Explanation of any interim analyses and stopping guidelines, including who will have access to these interim results and make the final decision to terminate the trial | No interim analyses or formal stopping guidelines were planned as the intervention is low risk, non-invasive, and not expected to cause harm. Study continuation is overseen through regular investigator meetings and institutional ethics monitoring |
| Trial monitoring | 29 | Frequency and procedures for monitoring trial conduct. If there is no monitoring, give explanation | No formal monitoring procedures were implemented as the trial involves a low-risk behavioural intervention delivered within existing home care services. Trial conduct is instead overseen through routine investigator review and ethics committee reporting requirements |
| **Ethics** | | | |
| Research ethics approval | 30 | Plans for seeking research ethics committee/institutional review board approval | Page 12, Lines 240 - 245 |
| Protocol amendments | 31 | Plans for communicating important protocol modifications to relevant parties | Not reported |
| Consent or assent | 32a | Who will obtain informed consent or assent from potential trial participants or authorised proxies, and how | Page 12, Lines 240 - 245 |
|  | 32b | Additional consent provisions for collection and use of participant data and biological specimens in ancillary studies, if applicable | Page 21, Lines 344 - 351 |
| Confidentiality | 33 | How personal information about potential and enrolled participants will be collected, shared, and maintained in order to protect confidentiality before, during, and after the trial | Page 21, Lines 344 - 3561 |
| Ancillary and post-trial care | 34 | Provisions, if any, for ancillary and post-trial care, and for compensation to those who suffer harm from trial participation | N/A to study design |

# Supplement 2

Structure Summary of trial design and methods, including items from the World Health Organisation Trial Registration Data Set

| Items | Response |
| --- | --- |
| Primary registry and trial identifying number | ANZCTR  ACTRN12624001167550 |
| Date of registration in primary registry | 25/09/2024 |
| Secondary identifying numbers | N/A |
| Universal trial number (UTN) | U1111-1329-2424 |
| Source(s) of monetary or material Support | NHMRC 2022 MRFF Dementia Ageing and Aged Care Mission 2024352 |
| Primary sponsor | The University of Queensland  Centre for Hearing Research  School of Health and Rehabilitation Sciences  St Lucia, QLD 4072 |
| Secondary sponsor(s) | N/A |
| Contact for public queries | Dr Melinda Toomey  Phone: +61 7 3443 6073  Email: m.toomey@uq.edu.au |
| Contact for scientific queries | Dr Melinda Toomey  Phone: +61 7 3443 6073  Email: m.toomey@uq.edu.au |
| Public title | Evaluating the impact of hearing and vision support in home care settings |
| Scientific title | Implementation and evaluation of a home hearing and vision care program to improve quality of life for frail older Australians |
| Countries of recruitment | Australia |
| Health condition(s) | Hearing impairment  Vision impairment |
| Intervention(s) | Sensory Support Intervention comprising:   1. Identify any vision and hearing concerns and discuss support options 2. Home-based functional assessment and goal setting 3. Optimisation of any vision or hearing impairment 4. Continuous training in correct use of sensory devices 5. Communication training 6. Referral to health and social services 7. Fostering social inclusion through hobbies/interests / social groups 8. Environmental modifications and assistive devices |
| Key inclusion and exclusion criteria | Inclusion criteria for older adults are (i) aged 65 years or older, (ii) cognitive status from normal to moderately-advanced dementia ( Functional Assessment Staging Test Score Stage 6), (iii) residing at home and receiving home care services (either Home Care Packages or Commonwealth Home Support Program); (iv) capacity to provide informed consent or have a care partner to provide proxy consent, (v) adult-acquired hearing impairment (pure tone audiometric thresholds worse than 20 dB HL at 1000Hz or 2000Hz, or worse than 35 dB HL, 4000Hz, or 8000Hz in the better hearing ear) and/or adult-acquired vision impairment (presenting monocular visual acuity of 6/12 or worse in the better eye).  Exclusion criteria include a cataract surgery scheduled during the intervention period. |
| Study type | Pre-post intervention |
| Date of first enrolment | 15/07/2024 |
| Sample size | 87 |
| Recruitment status | Recruiting |
| Primary outcome(s) | Change in Health Utilities Index Mark 3 (HUI3 ) and Quality of Life Australian Aged Care Consumer (QOL-ACC) from baseline to weeks 13 and 26 post commencement of intervention. |
| Key secondary outcomes | Change in Adult Social Care Outcomes Toolkit (SCT4), Questions from UK Biobank, Instrumental Activities of Daily Living Scale, National Eye Institute Visual Function Questionnaire 25 item (NEI VFQ-25), Revised Hearing Handicap Inventory for the Elderly Screening version, Geriatric Anxiety Inventory (GAI) Short Form, and Geriatric Depression Scale SF (GDS-5) from baseline to weeks 13 and 26 post commencement of intervention. |
| Ethics review | Status: Approved  Approval date: 07/03/2024  Contact: The University of Queensland Human Ethics Committee |
| Data sharing statement | What data will be shared: Deidentified data (participant demographics, screening results, questionnaire responses)  Data will be shared |
| ANZCTR, Australian New Zealand Clinical Trials Registry |  |

# Supplement 3

The TIDieR (Template for Intervention Description and Replication) Checklist*:

|  | **Item** | **Where located** |
| --- | --- | --- |
|  | **BRIEF NAME** |  |
| **1.** | Provide the name or a phrase that describes the intervention. | Sensory Support intervention |
|  | **WHY** |  |
| **2.** | Describe any rationale, theory, or goal of the elements essential to the intervention. | Introduction, pages 5 – 8  Intervention description pages 13 - 17 |
|  | **WHAT** |  |
| **3.** | Materials: Describe any physical or informational materials used in the intervention, including those provided to participants or used in intervention delivery or in training of intervention providers. Provide information on where the materials can be accessed (e.g. online appendix, URL). | Table 2, pages 15 -17 |
| **4.** | Procedures: Describe each of the procedures, activities, and/or processes used in the intervention, including any enabling or support activities. | Intervention description, pages 13 - 17 |
|  | **WHO PROVIDED** |  |
| **5.** | For each category of intervention provider (e.g. psychologist, nursing assistant), describe their expertise, background and any specific training given. | Page 14, Lines 306 - 308 |
|  | **HOW** |  |
| **6.** | Describe the modes of delivery (e.g. face-to-face or by some other mechanism, such as internet or telephone) of the intervention and whether it was provided individually or in a group. | Page 14, Lines 305 - 306 |
|  | **WHERE** |  |
| **7.** | Describe the type(s) of location(s) where the intervention occurred, including any necessary infrastructure or relevant features. | Page 14, Lines 305 - 306 |
|  | **WHEN and HOW MUCH** |  |
| **8.** | Describe the number of times the intervention was delivered and over what period of time including the number of sessions, their schedule, and their duration, intensity or dose. | Page 14, Lines 305 - 306 |
|  | **TAILORING** |  |
| **9.** | If the intervention was planned to be personalised, titrated or adapted, then describe what, why, when, and how. | Page 5, Lines 308 -316 |
|  | **MODIFICATIONS** |  |
| **10.^ǂ^** | If the intervention was modified during the course of the study, describe the changes (what, why, when, and how). | N/A as we are reporting the protocol |
|  | **HOW WELL** |  |
| **11.** | Planned: If intervention adherence or fidelity was assessed, describe how and by whom, and if any strategies were used to maintain or improve fidelity, describe them. | Not reported in this protocol. Reported in the Process Evaluation protocol. |
| **12.^ǂ^** | Actual: If intervention adherence or fidelity was assessed, describe the extent to which the intervention was delivered as planned. | N/A as we are reporting the protocol. |

Hróbjartsson, A., I. Boutron, S. Hopewell, D. Moher, K. F. Schulz, G. S. Collins, R. Tunn, R. Aggarwal, M. Berkwits, J. A. Berlin, N. Bhandari, N. J. Butcher, M. K. Campbell, R. C. W. Chidebe, D. R. Elbourne, A. J. Farmer, D. A. Fergusson, R. M. Golub, S. N. Goodman, T. C. Hoffmann, J. P. A. Ioannidis, B. C. Kahan, R. L. Knowles, S. E. Lamb, S. Lewis, E. Loder, M. Offringa, P. Ravaud, D. P. Richards, F. W. Rockhold, D. L. Schriger, N. L. Siegfried, S. Staniszewska, R. S. Taylor, L. Thabane, D. J. Torgerson, S. Vohra, I. R. White and A. W. Chan (2025). "SPIRIT 2025 explanation and elaboration: updated guideline for protocols of randomised trials." Bmj **389**: e081660.
